# Mediatory roles of leukotriene B_4_ receptors in LPS-induced endotoxic shock

**DOI:** 10.1038/s41598-019-42410-8

**Published:** 2019-04-11

**Authors:** Sun-Young Kwon, MyungJa Ro, Jae-Hong Kim

**Affiliations:** Department of Biotechnology, College of Life Sciences, Korea University, Seoul, 02841 Korea

## Abstract

Sepsis, a systemic inflammatory response syndrome caused by infection, is the most common disease in patients treated in intensive care units. Endotoxic shock, the most critical form of sepsis, is caused by gram-negative bacterial infection. However, the detailed mechanism of endotoxic shock remains unclear. In the present study, we observed that the production of leukotriene B_4_ (LTB_4_) and 12(*S*)-hydroxyeicosatetraenoic acid (HETE), inflammatory lipid mediators acting on LTB_4_ receptors (BLT1 and BLT2), was significantly upregulated in peritoneal lavage fluid (PF) and serum from an LPS-induced endotoxic shock mouse model. Furthermore, BLT1/2-dependent signaling pathways mediated the expression of IL-17, IL-6, and IL-1β, key cytokines for the development of endotoxic shock, via NF-κB activation in the LPS-induced endotoxic shock mouse model. Additionally, inhibition of BLT1/2 significantly attenuated inflammation and tissue damage associated with endotoxic shock and enhanced the survival rate of mice with this inflammatory complication. Together, these results suggest that LTB_4_ receptors play critical mediatory roles in the development of endotoxic shock. Our findings point to LTB_4_ receptors as potential therapeutic targets for the treatment of endotoxic shock.

## Introduction

Sepsis is a life-threatening condition caused by a dysregulated immune response to infection^1^. Endotoxic shock, the most severe complication of sepsis, is mainly caused by gram-negative bacterial infection and is defined as sepsis with organ dysfunction and hypotension^2–4^. Lipopolysaccharide (LPS), a major component of gram-negative bacteria, has been studied as a key mediator of the pathogenesis of bacterial infection, and it plays a pivotal role in endotoxic shock^3,5^. Endotoxic shock treatment includes antimicrobial therapy, source control, and intravenous immunoglobulin therapy^1,6^. Despite these medical efforts, endotoxic shock still carries a high risk of death and represents an economic burden. Thus, the identification of the signaling mechanisms underlying the development of endotoxic shock is a key for the discovery of interventions to reduce the associated mortality.

LPS-mediated signaling transduction pathways have been shown to stimulate the production of proinflammatory cytokines such as IL-17, IL-6, and IL-1β in endotoxic shock^7^. The high levels of these proinflammatory cytokines contribute to the induction of endotoxic shock^8–14^. For example, IL-17 is associated with the recruitment of neutrophils, IL-6 is associated with inflammatory tissue injury, and IL-1β is associated with tachycardia or hypotension^11,15,16^. Although IL-17, IL-6, and IL-1β play essential roles in endotoxic shock, the detailed mechanisms of their production remain unclear.

Leukotriene B_4_ (LTB_4_) is a potent proinflammatory lipid mediator derived from arachidonic acid by 5-lipoxygenase (5-LO)^17,18^. One of the major activities of LTB_4_ is the recruitment of leukocytes, including neutrophils^18^. LTB_4_ exerts its biological functions through two G protein-coupled receptors (GPCRs), BLT1 and BLT2^19^. BLT1, a high-affinity receptor for LTB_4_, is mainly expressed in inflammatory cells, such as leukocytes. Unlike BLT1, the low-affinity LTB_4_ receptor BLT2 is expressed in a variety of tissues^20^. In contrast to the relatively specific binding of LTB_4_ to BLT1, several arachidonic acid metabolites such as 12(*S*)-hydroxyeicosatetraenoic acid (HETE) and 12-hydroxyheptadecatrienoic acid (12-HHT) have also been identified as ligands for BLT2^21^. Previous reports suggest that 5-LO, the enzyme catalyzing LTB_4_ production, is associated with endotoxic conditions^22,23^. Previous studies have shown that BLT1 and BLT2 are associated with LPS-mediated inflammatory signaling pathways^24,25^. Based on these reports, we investigated whether BLT1/2 cascades play any role in the development of endotoxic shock.

In the current study, we found that BLT1/2 contribute to IL-17, IL-6, and IL-1β production through NF-κB activation in LPS-induced endotoxic shock. In addition, we observed that blockade of BLT1/2 attenuates LPS-induced endotoxic inflammation and tissue damage and improves the survival rate. Therefore, these results suggest that LTB_4_ receptors play critical mediatory roles in the development of endotoxic shock. Our findings point to BLT1/2 as potential therapeutic targets for the treatment of endotoxic shock.

## Materials and Methods

### Reagents

LPS (*Escherichia coli* serotype O55:B5) and dimethyl sulfoxide (DMSO) were obtained from Sigma-Aldrich (St Louis, MO). The BLT1 antagonist U75302 and the 12-LO inhibitor Baicalein were obtained from Enzo Life Sciences (Farmingdale, NY). The BLT2 antagonist LY255283 was obtained from Cayman Chemical (Ann Arbor, MI). The 5-LO inhibitor MK886 and the NF-κB inhibitor Bay11-7082 were obtained from Calbiochem (La Jolla, CA).

### Endotoxic shock mouse model

Male C57BL/6N mice (8–10 weeks old) were obtained from the Experimental Animal Center of Korea University. At 8 weeks of age, these mice were distributed into the following groups: (A) Normal group; (B) LPS group, in which the mice were intraperitoneally injected with vehicle (DMSO); (C) U75302 (500 μg/kg) + LPS group; (D) LY255283 (10 mg/kg) + LPS group; (E) MK886 (1 mg/kg) + LPS group; (F) Baicalein (20 mg/kg) + LPS group; and (G) Bay11-7082 (5 mg/kg) + LPS group. All mice (except those in the normal group) were intraperitoneally injected with LPS (10 mg/kg) dissolved in PBS to induce endotoxic shock for 6 h. After the injection of LPS (50 mg/kg), mice (9–10 weeks old) were monitored for survival over 120 h. All animals were housed under 12:12 h light/dark conditions with three to five mice per static polycarbonate microisolator cage on disposable bedding. Wire-lidded food hoppers within the cages were filled to capacity with rodent chow, and the mice had access to bottle-supplied water. All experimental animals used in this study were treated according to guidelines approved by the Institutional Animal Care and Use Committee of Korea University.

### Measurement of IL-17, IL-6, IL-1β, LTB_4_ and 12(*S*)-HETE

Six hours after LPS injection, peritoneal lavage fluid (PF) samples were taken from mice via the abdominal cavity. Blood samples were obtained from mice through the right ventricle orbital veniplex. The collected PF was centrifuged at 6000 × *g* for 5 min at 4 °C, and the blood was centrifuged at 18000 × *g* for 15 min at room temperature (RT). The supernatants were stored at −80 °C until they were assayed for cytokines and mediators. The levels of IL-17, IL-1β (R&D systems, Minneapolis, MN), and IL-6 (BD Biosciences, San Diego, CA) in PF and serum were quantified using Enzyme-linked immunosorbent assay (ELISA) kits according to the manufacturer’s instructions. The levels of LTB_4_ and 12(*S*)-HETE (Enzo Life Sciences, Farmingdale, NY) in PF and serum were determined by the use of ELISA kits according to the manufacturer’s recommendations.

### Preparation of lung tissue lysates and immunoblot analysis

Homogenized lung tissue was lysed in lysis buffer (50 mM Tris-Cl (pH 7.4), 150 mM NaCl, 5 mM EDTA, 0.1% Triton X-100, 0.02% NaN_3_, 100 mM phenylmethylsulfonyl fluoride, 1 M sodium fluoride, leupeptin (0.5 mg/ml), aprotinin (1 mg/ml), and pepstatin A (5 mg/ml)) at 4 °C and then heated at 95 °C for 5 min. The lysates were then subjected to SDS-PAGE, and the separated proteins were transferred electrophoretically to a polyvinylidenedifluoride (PVDF) membrane for 1 h at 100 V. The membrane was incubated overnight at 4 °C with TBST containing 5% nonfat dried milk and at RT for 1 h with primary antibodies at a 1:1000 dilution (1:500 for BLT1 and BLT2 or 1:2000 for β-actin) in TBST containing 5% nonfat dried milk. The membrane was then incubated for 1 h at RT with horseradish peroxidase (HRP)-linked secondary antibodies before the detection of immune complexes with an enhanced chemiluminescence kit (GE Healthcare Life Sciences, Little Chalfont, UK). Proteins were quantified using ImageJ software (NIH, Bethesda, MD).

### Flow cytometry analysis

Six hours after LPS injection, PF was harvested by lavage of the mouse abdominal cavity with 2 ml cold PBS. The collected PF was centrifuged at 6000 × *g* for 5 min at 4 °C. The supernatants were stored at −80 °C before cytokine levels were measured, and the pellets were resuspended. The peritoneal cells were incubated with FITC-conjugated anti-mouse Ly-6G and PE-conjugated anti-mouse CD11b (eBioscience, San Diego, CA) in FACS buffer (PBS containing 2% FBS) for 20 min. After being washed three times, the peritoneal cells were suspended in FACS buffer. Total neutrophils in PF were detected by flow cytometry (FACS Accuri; BD Biosciences, Franklin Lakes, NJ) and were gated on Ly-6G and CD11b to ascertain the percentage of neutrophils in PF.

### Histological examination

Excised lung and liver tissues were fixed in 3% formaldehyde for 3 weeks, dehydrated with increasing concentrations of ethanol, embedded in paraffin then cut into 6 μm sections. The paraffin sections were deparaffinized, hydrated and stained with hematoxylin and eosin (H&E) for histological examinations. All images were acquired using a BX51 microscope (Olympus, Tokyo, Japan) equipped with a DP71 digital camera (Olympus, Tokyo, Japan). Tissue injury score was determined in a blinded test on a semiquantitative scale ranging from 0 (normal) to 3 (severe).

### Statistical analysis

Data are reported as means ± standard deviation (SD) for three replicate experiments. Data were evaluated by one-way analysis of variance (ANOVA). Post hoc comparisons were made with Bonferroni’s test. The log rank test was applied to the survival data. Differences with *p* < 0.05 were considered statistically significant. Values of *p* < 0.05, *p* < 0.01 and *p* < 0.001 are designated by ^*^, ^**^ and ^***^, respectively.

## Results

### Leukotriene B_4_ receptors mediate IL-17, IL-6, and IL-1β synthesis in an LPS-induced endotoxic shock mouse model

**Figure 1:**
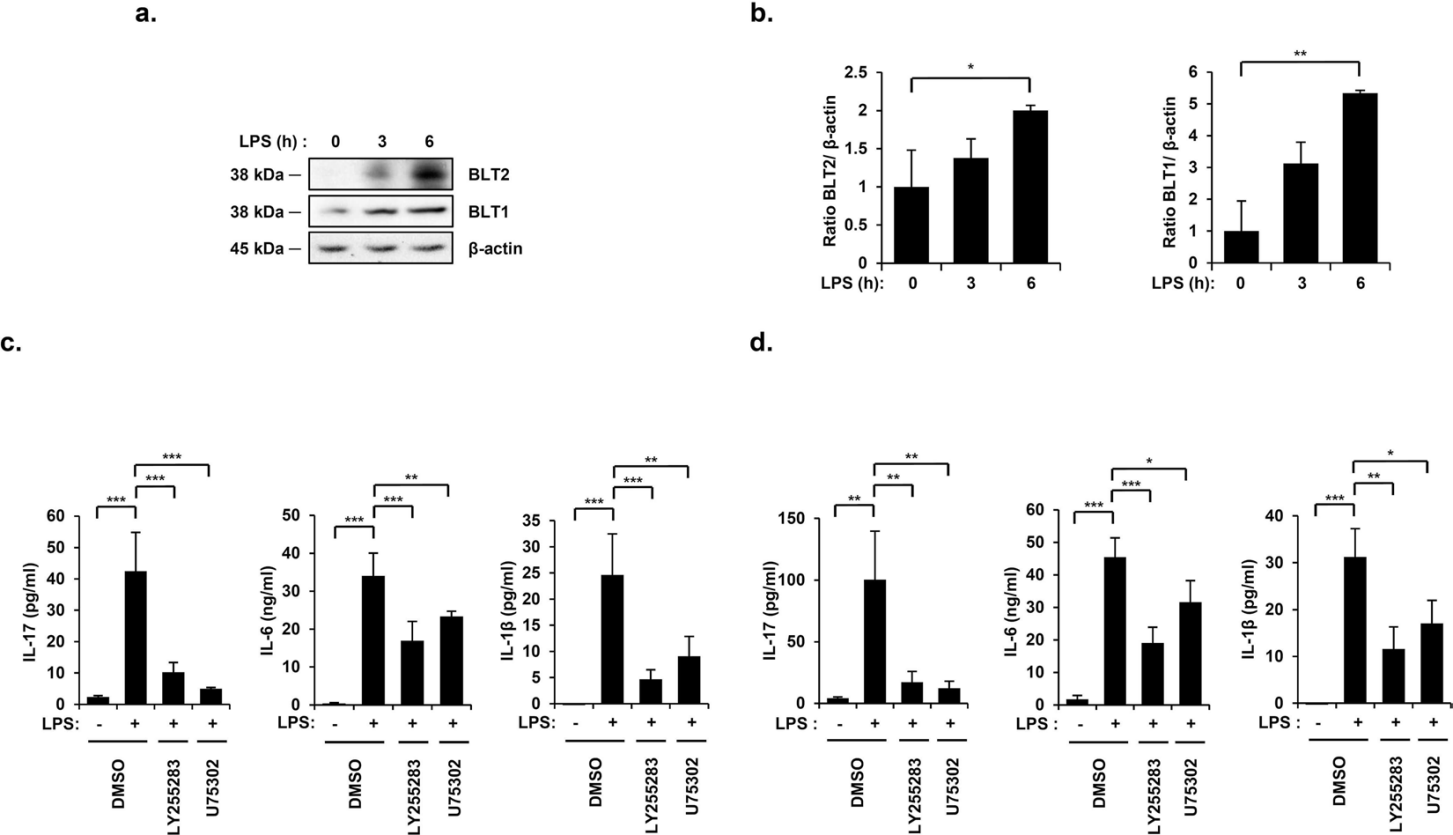
Leukotriene B_4_ receptors mediate IL-17, IL-6, and IL-1β synthesis in an LPS-induced endotoxic shock mouse model. Mice were intraperitoneally injected with LPS (10 mg/kg) for the indicated time periods, and lung tissues were harvested. (**a**) The mouse lung tissues were homogenized, and the protein levels of BLT1 and BLT2 were assessed by immunoblot assay. (38 kDa, BLT1; 38 kDa, BLT2; 45 kDa, β-actin) (**b**) Quantification of BLT1 and BLT2 levels. Data are representative of three independent experiments with similar results. *p < 0.05, **p < 0.01 versus the control group. Mice were intraperitoneally administered U75302 (500 μg/kg) or LY255283 (10 mg/kg) 1 h before LPS injection, and PF and blood were collected. (**c**) The amount of IL-17, IL-6, and IL-1β in PF was analyzed using specific ELISA kits. Data are shown as the mean ± SD (n = 3–6 per group). **p < 0.01, ***p < 0.001 versus each control group. (**d**) The serum levels of IL-17, IL-6, and IL-1β were analyzed using specific ELISA kits. Data are shown as the mean ± SD (n = 3–5 per group). *p < 0.05, **p < 0.01, ***p < 0.001 versus each control group.

Previous research showed that endotoxic shock causes a significant increase in serum LTB_4_ levels^26^. Thus, we investigated the potential roles of LTB_4_ receptors (BLT1 and BLT2) in IL-17, IL-6, and IL-1β production under conditions of LPS-induced endotoxic shock. First, we analyzed BLT1/2 levels in lungs and found that they were markedly upregulated in a time-dependent manner following LPS injection (Fig. 1a,b). To examine whether BLT1/2 contribute to IL-17, IL-6, and IL-1β production in an LPS-induced endotoxic shock mouse model, we pretreated mice with the BLT1 antagonist U75302 or the BLT2 antagonist LY255283 for 1 h before LPS injection. Then, we determined IL-17, IL-6, and IL-1β levels in PF and serum. Blockade of BLT1 or BLT2 significantly reduced the LPS-induced production of IL-17, IL-6, and IL-1β in PF and serum (Fig. 1c,d). Together, these results suggest that the LTB_4_ receptors BLT1 and BLT2 are essential for the production of IL-17, IL-6, and IL-1β in an LPS-induced endotoxic shock mouse model.

### 5-/12-LO lie upstream of BLT1/2 to mediate IL-17, IL-6, and IL-1β synthesis in an LPS-induced endotoxic shock mouse model

**Figure 2:**
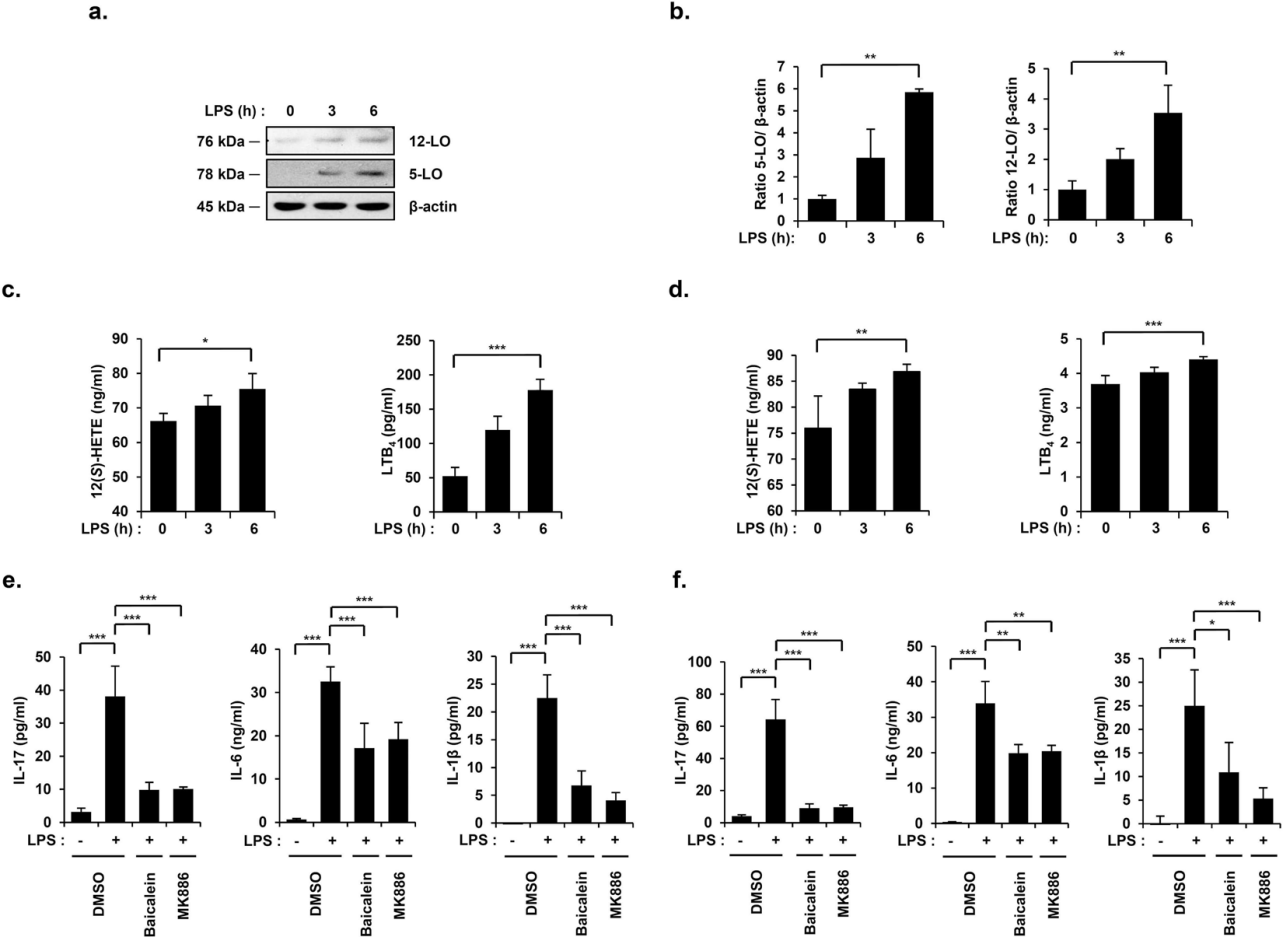
5-/12-LO lie upstream of BLT1/2 to mediate IL-17, IL-6, and IL-1β synthesis in an LPS-induced endotoxic shock mouse model. Mice were intraperitoneally injected with LPS (10 mg/kg) for the indicated time periods, and lung tissues, PF and blood were harvested. (**a**) The mouse lung tissues were homogenized, and the protein levels of 5-LO and 12-LO were assessed by immunoblot assay. (78 kDa, 5-LO; 76 kDa, 12-LO; 45 kDa, β-actin) (**b**) Quantification of 5-LO and 12-LO levels. Data are representative of three independent experiments with similar results. ^**^p < 0.01 versus the control group. (**c**) The levels of LTB_4_ and 12(*S*)-HETE in PF were analyzed using specific ELISA kits. Data are shown as the mean ± SD (n = 3–5 per group). ^*^p < 0.05, ^***^p < 0.001 versus the control group. (**d**) The serum levels of LTB_4_ and 12(*S*)-HETE were analyzed using specific ELISA kits. Data are shown as the mean ± SD (n = 3–5 per group). ^**^p < 0.01, ^***^p < 0.001 versus the control group. Mice were intraperitoneally administered MK886 (1 mg/kg) or Baicalein (20 mg/kg) 1 h before LPS injection, and PF and blood were collected. (**e**) The amount of IL-17, IL-6, and IL-1β in PF was analyzed using specific ELISA kits. Data are shown as the mean ± SD (n = 4–6 per group) ^***^p < 0.001 versus each control group. (**f**) The serum levels of IL-17, IL-6, and IL-1β were analyzed using specific ELISA kits. Data are shown as the mean ± SD (n = 3–6 per group). ^*^p < 0.05, ^**^p < 0.01, ^***^p < 0.001 versus each control group.

5-LO and 12-LO catalyze the synthesis of LTB_4_ and 12(*S*)-HETE from arachidonic acid^17,27^. Thus, we tested whether 5-/12-LO play any roles in LPS-induced endotoxic shock. We found that the protein expression levels of 5-LO and 12-LO in lungs were markedly increased in a time-dependent manner following LPS injection (Fig. 2a,b). We also observed that LTB_4_ and 12(*S*)-HETE levels in PF and serum increased in a time-dependent manner following LPS injection (Fig. 2c,d). To examine whether 5-/12-LO contribute to IL-17, IL-6, and IL-1β production in an LPS-induced endotoxic shock mouse model, we pretreated mice with the 5-LO inhibitor MK886 or the 12-LO inhibitor Baicalein for 1 h before LPS injection. Blockade of 5-LO or 12-LO significantly reduced the LPS-induced production of IL-17, IL-6, and IL-1β in PF and serum (Fig. 2e,f). Taken together, these results suggest that 5-/12-LO, lying upstream of BLT1/2, mediate IL-17, IL-6, and IL-1β production in an LPS-induced endotoxic shock mouse model.

### NF-κB lies downstream of 5-/12-LO-BLT1/2 cascades to mediate IL-17, IL-6, and IL-1β synthesis in an LPS-induced endotoxic shock mouse model

**Figure 3:**
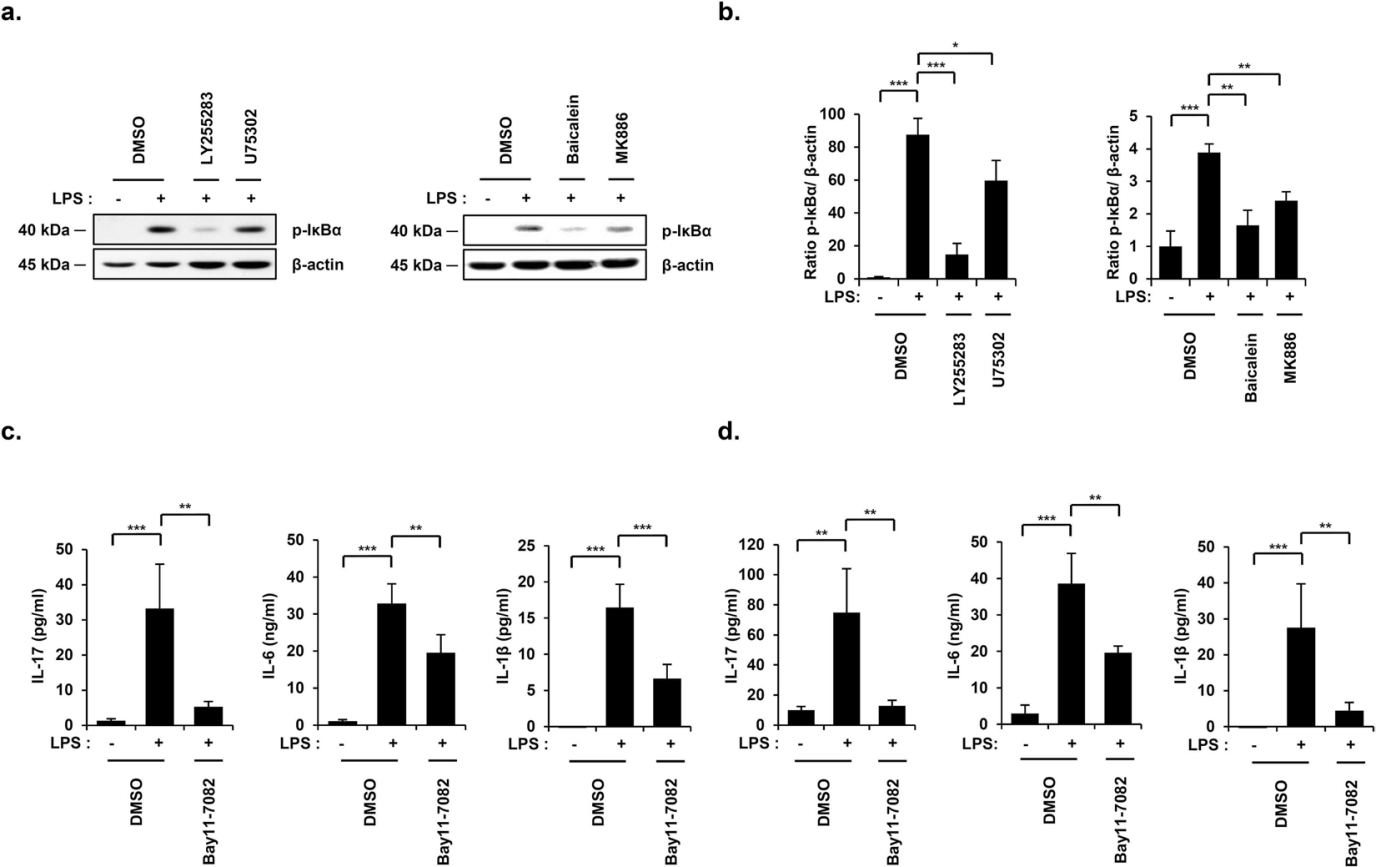
NF-κB lies downstream of 5-/12-LO-BLT1/2 cascades to mediate IL-17, IL-6, and IL-1β synthesis in an LPS-induced endotoxic shock mouse model. Mice were intraperitoneally administered U75302 (500 μg/kg), LY255283 (10 mg/kg), MK886 (1 mg/kg) or Baicalein (20 mg/kg) 1 h before LPS injection, and lung tissues, PF and blood were harvested. (**a**) The mouse lung tissues were homogenized, and the protein levels of p-IκBα were assessed by immunoblot assay. (40 kDa, p-IκBα; 45 kDa, β-actin) (**b**) Quantification of p-IκBα levels. Data are representative of three independent experiments with similar results. ^*^p < 0.05, ^**^p < 0.01, ^***^p < 0.001 versus each control group. Mice were intraperitoneally administered Bay11-7082 (5 mg/kg) 1 h before LPS injection, and PF and blood were collected. (**c**) The amount of IL-17, IL-6, and IL-1β in PF was analyzed using specific ELISA kits. Data are shown as the mean ± SD (n = 3–5 per group). ^**^p < 0.01, ^***^p < 0.001 versus each control group. (**d**) The serum levels of IL-17, IL-6, and IL-1β were analyzed using specific ELISA kits. Data are shown as the mean ± SD (n = 3–5 per group). ^**^p < 0.01, ^***^p < 0.001 versus each control group.

Previous studies demonstrated that BLT1/2 mediate LPS-induced NF-κB activation in macrophages^24,25^. We investigated whether 5-/12-LO-BLT1/2 cascades mediate IL-17, IL-6, and IL-1β production by activating NF-κB in an LPS-induced endotoxic shock. We pretreated mice with U75302, LY255283, MK886 or Baicalein for 1 h before LPS injection and observed a significant attenuation of NF-κB activation, as indicated by the reduced protein expression levels of p-IκBα in LPS-treated lungs (Fig. 3a,b). In addition, we examined whether NF-κB contribute to IL-17, IL-6, and IL-1β production in an LPS-induced endotoxic shock mouse model, we pretreated mice with the NF-κB inhibitor Bay11-7082 for 1 h before LPS injection. Blockade of NF-κB with Bay11-7082 significantly reduced the production of IL-17, IL-6, and IL-1β in PF and serum (Fig. 3c,d). Together, these results suggest that 5-/12-LO-BLT1/2 cascades mediate the production of IL-17, IL-6, and IL-1β by activating NF-κB in an LPS-induced endotoxic shock mouse model.

### Blockade of BLT1/2 suppresses inflammation in an LPS-induced endotoxic shock mouse model

**Figure 4:**
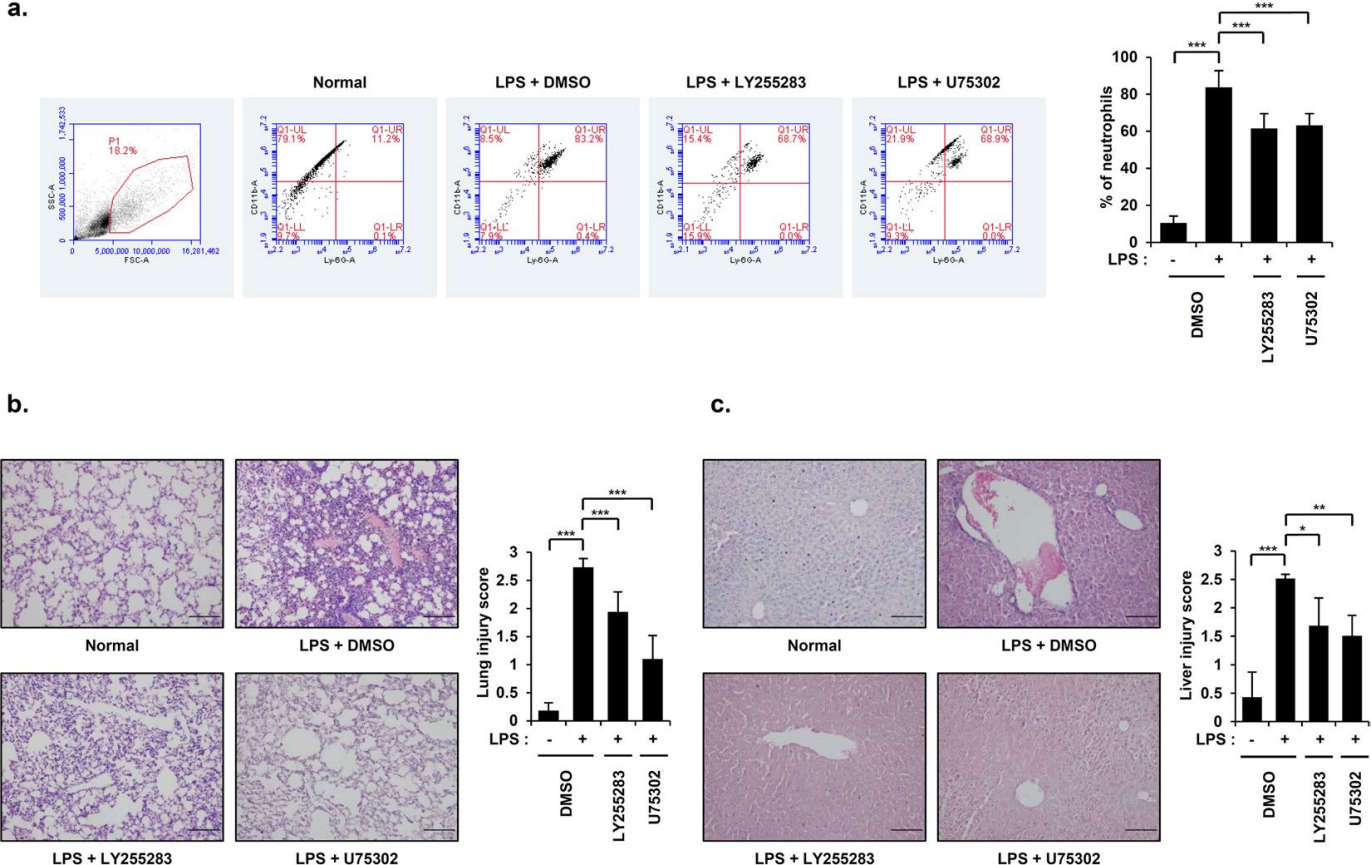
Blockade of BLT1/2 suppresses inflammation in an LPS-induced endotoxic shock mouse model. Mice were intraperitoneally administered U75302 (500 μg/kg) or LY255283 (10 mg/kg) 1 h before LPS injection. Six hours after LPS injection, PF, lung and liver tissues were obtained from mice. (**a**) Percentage of neutrophils in PF. Neutrophils were detected by flow cytometry. Representative FACS plots of neutrophils (CD11b^+^Ly6G^+^ cells) in vehicle-treated, U75302-treated and LY255283-treated mice in PF collected at 6 h postinjection with LPS. Data are shown as the mean ± SD (n = 5–12 per group). ^***^p < 0.001 versus each control group. (**b**) Representative lung histology (*left*) and lung injury score (*right*) from the indicated groups. Data are shown as the mean ± SD (n = 7 per group). ^***^p < 0.001 versus each control group. (**c**) Representative liver histology (*left*) and liver injury score (*right*) from the indicated groups. Data are shown as the mean ± SD (n = 7 per group). ^*^p < 0.05, ^**^p < 0.01, ^***^p < 0.001 versus each control group. Scale bar, 50 μm.

Neutrophils play a pivotal role in the development of organ dysfunction, a hallmark of endotoxic shock^28^. Next, we investigated whether the inhibition of BLT1/2 attenuates the recruitment of neutrophils and tissue damage. Thus, we pretreated mice with U75302 or LY255283 for 1 h before LPS injection and observed significantly suppressed inflammation and attenuated neutrophil recruitment in PF (Fig. 4a). Lung and liver tissue sections were prepared for histological analysis by H&E staining. Compared with the control group, the LPS-treated group showed alveolar hemorrhage and an influx of immune cells in lung tissues. LPS-treated liver tissues showed necrosis and leukocyte infiltration into the parenchyma. In contrast, pretreatment with the abovementioned inhibitors markedly attenuated inflammation and tissue damage induced by endotoxic shock (Fig. 4b,c). Taken together, these results suggest that the inhibition of BLT1/2 suppresses inflammation in an LPS-induced endotoxic shock mouse model.

### Blockade of BLT1/2 extends the survival of mice with LPS-induced endotoxic shock

**Figure 5:**
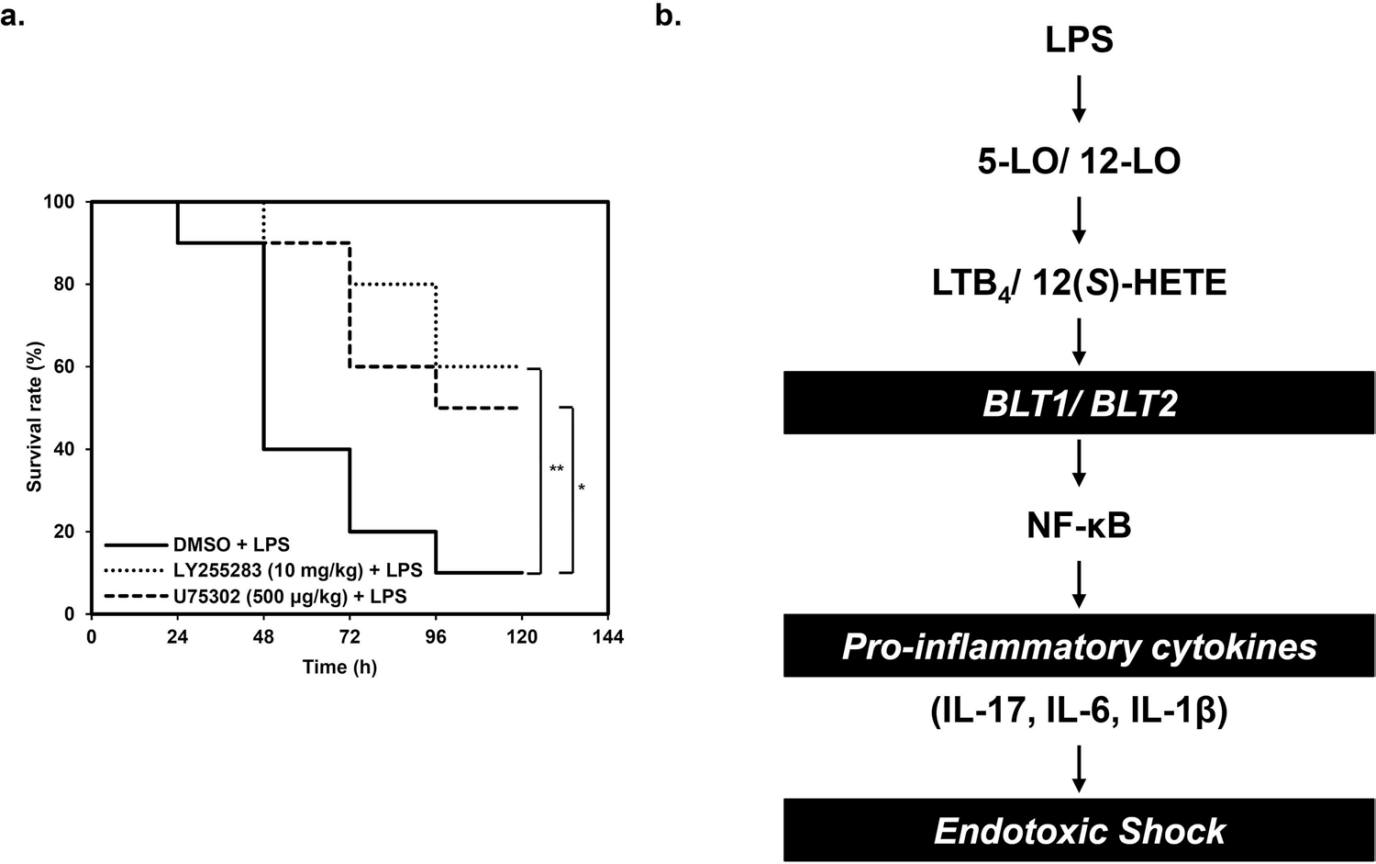
Blockade of BLT1/2 extends the survival of mice with LPS-induced endotoxic shock. Mice were intraperitoneally administered U75302 (500 μg/kg) or LY255283 (10 mg/kg) 1 h before LPS injection (50 mg/kg), and mice were monitored for survival over 120 h. (**a**) The percentage of survival was evaluated according to the log rank test, and the difference among experimental groups was significant. Data are shown as the mean ± SD (n = 10 per group). ^*^p < 0.05 and ^**^p < 0.01 compared to the control group. (**b**) Scheme of 5-/12-LO-BLT1/2 signaling cascades in an LPS-induced endotoxic shock mouse model.

To investigate whether BLT1/2 affect the survival rate of mice with LPS-induced endotoxic shock, we pretreated mice with U75302 or LY255283 for 1 h before LPS injection and then induced endotoxic shock with LPS. The inhibition of BLT1/2 increased the survival rate compared with LPS treatment alone (Fig. 5a). These results suggest that BLT1/2 contribute to extending the survival rate with LPS-induced endotoxic shock, further supporting the proinflammatory roles of BLT1/2 in LPS-induced endotoxic shock (Fig. 5b).

## Discussion

In the current study, we found that 5-/12-LO-BLT1/2 cascades mediate IL-17, IL-6, and IL-1β production in an LPS-induced endotoxic shock mouse model. In addition, we determined that NF-κB, acting downstream of BLT1/2, mediates the LPS-induced production of IL-17, IL-6, and IL-1β. Taken together, our findings suggest that 5-/12-LO-BLT1/2 cascades stimulate IL-17, IL-6, and IL-1β production via NF-κB activation, thus potentially contributing to the development of LPS-induced endotoxic shock.

LPS-induced endotoxic shock was previously shown to be mediated by the enhanced production of IL-17, IL-6, and IL-1β, key proinflammatory cytokines in the development of endotoxic shock^8,13^. The critical roles of IL-6 and IL-1β in LPS-induced inflammation, such as occurs in endotoxic shock, are already known^7,13,16^, and IL-17 was recently reported to be involved in endotoxic inflammatory complications^8^. Interestingly, these cytokines have been reported to be involved in neutrophil influx, tissue injury, and hypotension in endotoxic shock^15,16^. Despite the reported roles of IL-17, IL-6, and IL-1β in the development of endotoxic shock, the detailed signaling mechanisms underlying their production remained incompletely elucidated. In the present study, we observed that the expression levels of BLT1/2 and 5-/12-LO, the enzymes catalyzing the production of the ligands for these receptors, were markedly increased in a time-dependent manner following LPS injection (Figs 1a and 2a) and that the inhibition of BLT1/2 or 5-/12-LO significantly reduced IL-17, IL-6, and IL-1β production in an LPS-induced endotoxic shock mouse model (Figs 1c,d and 2e,f).

Consistent with these results, a previous report showed that 5-LO, the enzyme catalyzing the production of LTB_4_, is associated with endotoxic shock^22,23^. LTB_4_, a product of 5-LO, has been shown to be increased in an LPS-induced endotoxic shock animal model, suggesting that it is potentially associated with endotoxic shock^22^. Unlike 5-LO, the role of 12-LO in endotoxic shock has not yet been studied. Our current study is the first report on the roles of 12-LO and 12(*S*)-HETE, as well as their receptor BLT2, in the development of LPS-induced endotoxic shock. We found that LPS upregulated the production of LTB_4_ and 12(*S*)-HETE in an LPS-induced endotoxic shock mouse model (Fig. 2c,d).

Moreover, LTB_4_ was shown to stimulate the production of proinflammatory cytokines, contributing to neutrophil influx and tissue damage under conditions of endotoxic shock^29^. Neutrophils have been suggested to play a pivotal role in multiple organ failure, a hallmark of endotoxic shock^28^. BLT1/2 inhibition significantly attenuated the recruitment of neutrophils in PF (Fig. 4a). Moreover, blockade of BLT1/2 suppressed the influx of immune cells in the lung and liver tissues and ameliorated inflammation (Fig. 4b,c), suggesting that BLT1/2 play pivotal roles in LPS-induced endotoxic shock. In addition, BLT1/2 inhibition enhanced the survival rate compared with LPS treatment alone (Fig. 5a). The previous study noted that the LPS model is similar to the cecal ligation puncture (CLP) model in terms of mortality, but they have significant differences in terms of the kinetics and magnitude of cytokine production^30^. Therefore, it would be better to carry out further studies with the CLP model which seems to be closer to actual clinically seen patient states.

Previous studies have suggested that NF-κB lies downstream of BLT1/2 and that NF-κB activation is essential for the development of endotoxic shock^24,25,31^. Based on these reports, we examined whether NF-κB, lying downstream of 5-/12-LO-BLT1/2 cascades, regulates IL-17, IL-6, and IL-1β production to induce endotoxic shock. The inhibition of 5-/12-LO-BLT1/2 cascades significantly reduced IκBα phosphorylation in LPS-induced endotoxic shock (Fig. 3a), and the LPS-induced production of proinflammatory cytokines, including IL-17, IL-6, and IL-1β, was suppressed upon NF-κB inhibition (Fig. 3c,d). These findings suggest that 5-/12-LO-BLT1/2-NF-κB cascades regulate IL-17, IL-6, and IL-1β production in LPS-induced endotoxic shock.

In summary, our results demonstrate that LPS stimulates 5-/12-LO-BLT1/2 cascades and induces endotoxic shock by significantly increasing IL-17, IL-6, and IL-1β production via NF-κB (Fig. 5b). The identification of this mechanism suggests novel potential targets for the treatment of inflammatory complications such as endotoxic shock.

## Data Availability

The datasets generated during and/or analyzed during the current study are available from the corresponding author on reasonable request.
